# The Mast Cell–Substance P Neuroimmune Axis in Allergic Contact Dermatitis and Atopic Dermatitis: Molecular Mechanisms and Pathophysiological Perspectives

**DOI:** 10.3390/cimb48090928

**Published:** 2026-09-10

**Authors:** Ernesto Aitella, Gianluca Azzellino, Ciro Romano, Massimo De Martinis, Lia Ginaldi

**Affiliations:** 1 Department of Life, Health and Environmental Sciences, University of L’Aquila, 67100 L’Aquila, Italy; gianluca.azzellino@graduate.univaq.it (G.A.); massimomariamarcello.demartinis@univaq.it (M.D.M.); 2 Allergy and Clinical Immunology Unit, Center for the Diagnosis and Treatment of Osteoporosis, Azienda Unità Sanitaria Locale 04 Teramo, 64100 Teramo, Italy; 3 Department of Autoimmune Diseases, Hospital Clinic, University of Barcelona, 08036 Barcelona, Spain; 4 Complex Operational Unit, Adriatic District Area, Azienda Unità Sanitaria Locale 04 Teramo, 64100 Teramo, Italy; 5 Clinical Immunology Outpatient Clinic, Division of Internal Medicine, Department of Advanced Medical and Surgical Sciences, “Luigi Vanvitelli” University of Campania, 80131 Naples, Italy; ciro.romano@unicampania.it; 6 Long-Term Care Unit, “Maria SS. dello Splendore” Hospital, Azienda Unità Sanitaria Locale 04 Teramo, 64021 Teramo, Italy; 7 UniCamillus-Saint Camillus International University of Health Sciences, 00131 Rome, Italy

**Keywords:** mast cells, substance P, MRGPRX2, neuroimmune crosstalk, allergic contact dermatitis, atopic dermatitis, delayed-type hypersensitivity, neurogenic inflammation, chronic pruritus, inflammatory dermatitis

## Abstract

Recent advances in cutaneous neuroimmunology have substantially expanded our understanding of inflammatory skin diseases, revealing an intricate bidirectional network linking peripheral sensory neurons, resident immune cells, and structural skin components. Among the mediators orchestrating this communication, substance P (SP) and mast cells have emerged as pivotal regulators connecting neuronal activation with immune responses, vascular dysfunction, chronic inflammation, and persistent pruritus. Beyond the canonical neurokinin-1 receptor (NK1R), the identification of the Mas-related G protein-coupled receptor X2 (MRGPRX2) has fundamentally reshaped mast-cell biology by establishing an IgE-independent pathway of neuropeptide-induced activation. This narrative review examines the molecular mechanisms underlying the mast cell–SP neuroimmune axis and discusses its contribution to the pathogenesis of allergic contact dermatitis and atopic dermatitis. Unlike the traditional approach, which primarily considers atopic dermatitis as the reference model for cutaneous neuroimmune interactions, allergic contact dermatitis may provide a useful model for examining how neuroimmune amplification integrates with delayed T-cell-mediated inflammation. These concepts are subsequently applied to atopic dermatitis, where they operate within the broader context of type 2 inflammation, epidermal barrier dysfunction, and chronic pruritus. Rather than replacing classical immunopathogenic models, this emerging neuroimmune perspective complements them by identifying bidirectional communication between sensory neurons and mast cells as a dynamic amplifier of adaptive immune responses. This integrated perspective not only provides a unifying interpretation of inflammatory dermatitis but also offers a conceptual basis for exploring similar neuroimmune mechanisms across other immune-mediated skin disorders and for generating future mechanism-based therapeutic hypotheses.

## 1. Introduction

Peripheral nerves actively participate in inflammatory responses, and recent advances in neuroimmunology have substantially clarified the molecular mechanisms governing bidirectional communication between sensory neurons and resident immune cells. Among barrier organs, the skin represents one of the most specialized neuroimmune interfaces, where sensory nerve endings, epithelial cells, vascular structures, and tissue-resident immune cells coexist with a highly organized microenvironment that continuously integrates environmental, microbial, and inflammatory signals [1]. Within this dynamic microenvironment, even minor perturbations induced by physical stimuli, tissue injury, or microbial products can rapidly activate coordinated neuroimmune responses, amplifying local inflammation and, in susceptible individuals, contributing to the development and persistence of chronic inflammatory skin diseases [2].

Traditionally, neurogenic inflammation has been defined as the inflammatory response initiated by activation of peripheral sensory nerve fibers and the subsequent release of neuropeptides that promote vasodilation, increase vascular permeability, and facilitate immune cell recruitment [3]. However, this classical definition has progressively evolved. Neurogenic inflammation is now recognized as a complex network of reciprocal interactions in which neuronal mediators, resident immune cells, vascular components, and structural skin cells continuously influence one another, generating self-amplifying inflammatory circuits rather than isolated signaling events [4].

From a clinical perspective, this evolving view suggests that persistent inflammation, pruritus, and recurrent flares may reflect not only immune dysregulation but also sustained neuroimmune amplification, potentially identifying additional mechanisms of therapeutic relevance [5]. Although adaptive immune responses remain central to the pathogenesis of inflammatory dermatitis, accumulating evidence suggests that persistent bidirectional communication between sensory neurons and resident immune cells acts as a biological amplifier, sustaining inflammation, promoting chronic pruritus, and contributing to disease persistence. This integrated neuroimmune perspective provides a more comprehensive basis for understanding inflammatory dermatitis, particularly allergic contact dermatitis (ACD) and atopic dermatitis (AD), in which neuronal activation actively contributes to both disease initiation and progression [6].

Among the immune cells participating in this bidirectional communication, mast cells have emerged as central orchestrators of cutaneous neuroimmune signaling. Owing to their strategic localization at neurovascular interfaces and their remarkable functional plasticity, these long-lived, granule-rich immune cells reside in close proximity to peripheral sensory nerve endings and dermal blood vessels, enabling them to rapidly translate environmental and neuronal stimuli into coordinated inflammatory responses [7].

Substance P (SP) represents one of the most extensively investigated neuropeptides involved in cutaneous neuroimmune communication and has emerged as a pivotal molecular mediator linking neuronal activation to mast-cell effector functions. Although originally characterized as a tachykinin released by nociceptive sensory neurons, SP is now known to be produced by multiple immune and stromal cell populations, supporting its broader role as a multifunctional regulator of cutaneous inflammation rather than a simple neurotransmitter. The biological effects of SP were initially attributed almost exclusively to signaling through the neurokinin-1 receptor (NK1R), which is widely expressed by neuronal, immune, endothelial, and structural skin cells. Interestingly, the identification of MRGPRX2 as an additional SP-responsive receptor expressed on connective tissue mast cells has profoundly expanded our understanding of cutaneous neuroimmune communication. SP released from activated sensory neurons is a well-established mediator of neurogenic inflammation, acting on multiple peripheral target cells, including mast cells [8]. More recent experimental studies in human mast cells have shown that SP can induce rapid IgE-independent activation through MRGPRX2 [9]. However, it remains unclear to what extent these mechanisms are shared across inflammatory dermatoses with distinct immunological backgrounds and how the mast cell–SP axis contributes to their different pathogenic settings.

Against this background, the present review focuses specifically on the mast cell–SP axis as a bidirectional neuroimmune amplification system in allergic contact dermatitis and atopic dermatitis, with particular attention to NK1R- and MRGPRX2-mediated signaling. In contrast to our previous works, which addressed this axis within broader neuroimmune and inflammatory contexts, the present review adopts a disease-oriented comparative perspective within inflammatory dermatoses, specifically focusing on ACD and AD. We first summarize the molecular mechanisms underlying this interaction and then examine their contribution to both diseases. In particular, we explore the hypothesis that ACD may represent a mechanistically informative model for investigating neuroimmune amplification in delayed T-cell-mediated inflammation and assess to what extent these mechanisms may also contribute to the more complex immunopathology of AD. By comparatively examining these two inflammatory dermatoses, this review aims to identify shared neuroimmune mechanisms and disease-specific features, while highlighting unresolved pathophysiological and therapeutic questions.

### Literature Search Strategy and Study Selection

This narrative review was conducted to provide a comprehensive and up-to-date overview of the molecular mechanisms underlying the mast cell–SP neuroimmune axis and its contribution to the pathogenesis of allergic contact dermatitis and atopic dermatitis. A structured literature search was conducted in PubMed/MEDLINE, Scopus, and Web of Science covering publications from January 2000 to June 2026. Earlier seminal studies were also considered when identified through reference-list screening and deemed relevant to the historical or mechanistic context.

Search terms included combinations of “mast cells,” “substance P,” “MRGPRX2,” “neurokinin-1 receptor,” “NK1R,” “cutaneous neuroimmunology,” “neuroimmune crosstalk,” “neurogenic inflammation,” “pruritus,” “itch,” “allergic contact dermatitis,” “atopic dermatitis,” “delayed-type hypersensitivity,” “type 2 inflammation,” “mast cell activation,” and “inflammatory dermatitis”. Reference lists of relevant articles were also screened to identify additional studies.

Study selection was based on relevance to the scope of the review. Priority was given to original experimental and clinical studies addressing mast cell–SP interactions, NK1R/MRGPRX2 signaling, cutaneous neuroimmune communication, pruritus, and the pathophysiology of allergic contact dermatitis or atopic dermatitis. Reviews and meta-analyses were used to provide context and to identify additional primary studies. Titles and abstracts were screened for relevance, followed by full-text assessment when appropriate. Studies were excluded when they were unrelated to the main neuroimmune mechanisms addressed, provided limited mechanistic information, or focused on conditions outside the scope of the review.

Building on this literature, the present review critically integrates current evidence regarding the reciprocal interactions between mast cells and SP, with particular emphasis on NK1R- and MRGPRX2-mediated signaling in allergic contact dermatitis and atopic dermatitis. This conceptual perspective may also provide a foundation for investigating similar neuroimmune mechanisms across other immune-mediated skin disorders and for guiding the development of future precision medicine strategies.

## 2. Mast Cells as Key Effectors of Cutaneous Neuroimmune Communication

Mast cells derive from hematopoietic progenitors and complete their maturation after migrating into peripheral tissues, where local microenvironmental signals shape their final phenotype and functional profile [10]. This tissue-dependent maturation gives rise to heterogeneous mast cell populations with distinct protease content, receptor expression, activation thresholds, and mediator-release patterns. In the skin, mast cells are predominantly represented by connective tissue-type mast cells, which are enriched in the dermis and strategically localized around blood vessels, lymphatic vessels, hair follicles, and sensory nerve endings. This anatomical distribution places them at a strategic interface between the immune, vascular, epithelial, and nervous systems.

This tissue distribution also reflects functional mast-cell heterogeneity. Human mast cells are commonly distinguished into tryptase- and chymase-positive connective tissue-type mast cells (MC_TC) and predominantly tryptase-positive mucosal-type mast cells (MC_T) [11]. MRGPRX2 is preferentially expressed by MC_TC, making this population particularly responsive to SP and other cationic ligands [12,13]. However, MRGPRX2 expression is not uniform among cutaneous mast cells, suggesting that differences in receptor abundance and mast-cell phenotype may contribute to variability in neuropeptide responsiveness and, potentially, to variability in the effects of future MRGPRX2-targeted interventions [14,15].

The classical pathway of mast cell activation involves antigen-induced cross-linking of IgE bound to the high-affinity FcεRI receptor, resulting in rapid degranulation followed by the synthesis of lipid mediators, cytokines, and chemokines [16]. While this pathway remains essential for immediate allergic reactions, it does not fully account for the broader role of mast cells in chronic inflammatory and neuroimmune skin diseases. Increasing evidence indicates that mast cells can be activated through several IgE-independent mechanisms, thereby extending their function beyond classical allergy [17,18]. Mast cells express a broad repertoire of receptors for complement fragments, cytokines, chemokines, pattern-recognition signals, alarmins, and neuropeptides [19]. These receptors allow them to respond to microbial products, sterile tissue damage, endogenous danger signals, and changes in the local inflammatory microenvironment [20]. In addition, physical and environmental triggers such as pressure, friction, heat, cold, and emotional stress may influence mast cell activation, providing a biological explanation for flare-like phenomena observed in several chronic inflammatory skin disorders [21].

Among IgE-independent mechanisms of mast-cell activation, MRGPRX2 has emerged as a particularly relevant receptor in cutaneous mast cell biology. This receptor is prominently expressed by connective tissue mast cells and can be activated by several cationic ligands, including neuropeptides, host-defense peptides, antimicrobial peptides, and selected drugs [13]. Experimental studies have shown that SP can induce rapid IgE-independent degranulation of human mast cells through MRGPRX2, with evidence from cord blood-derived mast cells, the human LAD2 mast-cell line, and primary human skin mast cells [22,23]. Activation of MRGPRX2 induces rapid mast cell degranulation without requiring allergen-specific sensitization, thereby providing a mechanistic basis for pseudoallergic reactions and non-IgE-mediated inflammatory responses [24]. In the context of cutaneous neuroimmune communication, MRGPRX2 functions as a molecular gateway through which neuronal and stress-associated mediators can directly engage mast cell effector programs [12].

The biological relevance of mast cells in neuroimmune inflammation is amplified by their close anatomical relationship with peripheral sensory nerves [25]. Mast cells are frequently located near free nerve endings and nerve bundles, allowing bidirectional communication between these two cellular systems [26]. Following activation, mast cells orchestrate cutaneous inflammation through the rapid release of preformed mediators and the subsequent synthesis of cytokines, lipid mediators, chemokines, and growth factors that collectively regulate vascular responses, immune cell recruitment, tissue remodeling, and neuronal sensitization [27]. Preformed granule-associated molecules, including histamine, tryptase, chymase, heparin, and proteoglycans, are released within seconds to minutes after stimulation, promoting vasodilation, increased vascular permeability, extracellular matrix remodeling, leukocyte recruitment, and sensory nerve activation [28,29]. In parallel, activated mast cells synthesize a wide range of lipid mediators, cytokines, chemokines, and growth factors, including prostaglandins, leukotrienes, TNF-α, IL-4, IL-6, IL-13, IL-31, VEGF, and transforming growth factor-β, thereby sustaining inflammatory responses and contributing to tissue remodeling [30].

Beyond mediator release, mast cells actively communicate with multiple cellular components of the cutaneous microenvironment. Bidirectional interactions with keratinocytes, endothelial cells, fibroblasts, dendritic cells, eosinophils, neutrophils, and T lymphocytes contribute to the regulation of both innate and adaptive immune responses, highlighting the central role of mast cells in coordinating cutaneous inflammation rather than acting solely as terminal effector cells [31]. Of these interactions, the reciprocal communication between mast cells and peripheral sensory neurons has emerged as one of the key mechanisms underlying chronic inflammatory skin diseases. The close anatomical proximity between these two cell populations allows rapid exchange of molecular signals, whereby neuronal mediators activate mast cells and mast cell-derived products, in turn, modulate neuronal excitability, lowering the threshold for neuropeptide release and amplifying neuroimmune responses. This bidirectional communication establishes self-perpetuating inflammatory circuits that contribute to chronic pruritus, vascular dysfunction, and persistent tissue inflammation [8,32,33]. The recognition of mast cells as dynamic regulators of cutaneous neuroimmune communication has substantially expanded the traditional view of their biological function. Rather than being considered exclusively as effector cells of IgE-mediated hypersensitivity, mast cells are now regarded as multifunctional immune sentinels, integrating neuronal, epithelial, vascular, and immunological signals into coordinated inflammatory responses [34]. This updated view provides the biological basis for understanding the pathogenic relationship between SP, mast cell activation, chronic inflammation, and persistent itch in allergic and inflammatory skin diseases.

## 3. Substance P: Biology and Receptor-Mediated Signaling

SP, encoded by the TAC1 gene and primarily expressed by TRPV1-positive nociceptive sensory neurons, is an 11-amino acid neuropeptide belonging to the tachykinin family [35]. Owing to its evolutionary conservation and broad biological activity, SP has emerged as one of the principal mediators linking the nervous and immune systems, playing a central role in pain transmission, neurogenic inflammation, and stress-induced inflammatory responses [36]. Although traditionally regarded as a neurotransmitter released from peripheral sensory nerve endings, increasing evidence indicates that it can also be synthesized by several non-neuronal cell populations, including immune cells, keratinocytes, endothelial cells, and other tissue-resident cells [37]. This broader cellular distribution reinforces the concept of this neuropeptide as a multifunctional neuroimmune mediator capable of coordinating inflammatory responses within the cutaneous microenvironment.

The biological activity of SP is tightly controlled by its rapid extracellular degradation following release. Cell-surface peptidases, notably neprilysin, rapidly hydrolyze extracellular SP, resulting in a short tissue half-life and restricting its biological effects to the local microenvironment [38]. Consequently, its signaling primarily reflects localized release in close proximity to responsive target cells, emphasizing the importance of the anatomical relationship between sensory nerve endings and resident immune cells in the initiation and maintenance of neuroimmune responses.

The classical effects of SP are mediated through the neurokinin-1 receptor (NK1R), a G protein-coupled receptor widely expressed by neuronal, endothelial, epithelial, and immune cells, including mast cells [39]. Both full-length and truncated NK1R isoforms have been identified, which display distinct signaling properties that may influence receptor internalization, signal duration, and downstream inflammatory responses [40,41,42].

Upon ligand binding, NK1R activates phospholipase C, resulting in inositol trisphosphate-dependent intracellular calcium mobilization and protein kinase C activation, while simultaneously engaging MAPK and PI3K/Akt signaling pathways [43]. In addition, β-arrestin recruitment contributes to receptor internalization and prolonged intracellular signaling, ultimately promoting activation of transcription factors such as NF-κB and the subsequent production of pro-inflammatory cytokines and chemokines. Collectively, these signaling cascades enhance endothelial activation, leukocyte recruitment, vascular permeability, and the persistence of cutaneous inflammation [44,45].

However, NK1R does not fully explain the broad spectrum of SP-mediated inflammatory responses observed in the skin. In fact, the identification of MRGPRX2 has substantially expanded our current understanding of SP-mediated neuroimmune communication [8,46]. Highly expressed by connective tissue mast cells, MRGPRX2 mediates rapid, IgE-independent mast cell activation following stimulation by SP and several endogenous cationic peptides [47]. Unlike NK1R, which is broadly distributed among multiple cell types, MRGPRX2 provides a direct functional link between peripheral sensory neurons and mast cells, establishing one of the principal molecular mechanisms through which neuronal activation can rapidly trigger mast cell degranulation independently of allergen sensitization. The coexistence of these complementary signaling pathways therefore enables SP to coordinate inflammatory responses through distinct but complementary mechanisms, simultaneously modulating vascular, neuronal, epithelial, and immune functions while directly amplifying mast cell effector activity [9].

Importantly, human MRGPRX2 should be distinguished from its murine functional ortholog MrgprB2. Although both receptors mediate mast-cell responses to SP and other cationic ligands, differences in ligand specificity, expression patterns, and pharmacological responsiveness limit direct extrapolation from murine MrgprB2 studies to human MRGPRX2-mediated mechanisms [46].

Although this review primarily focuses on cutaneous neuroimmune interactions, it is important to recognize that persistent peripheral SP signaling may also contribute to central sensitization. Sustained nociceptive input originating from chronically inflamed tissues promotes enhanced glutamatergic neurotransmission within the spinal cord, facilitating long-lasting neuronal hyperexcitability and lowering pain thresholds [48]. Continuous activation of peripheral neuroimmune circuits may therefore reinforce central sensitization, providing a plausible biological explanation for the disproportionate pain and symptom burden observed in some chronic inflammatory disorders [49,50]. Nevertheless, the pathogenic role of SP in allergic and inflammatory skin diseases is primarily related to its capacity to establish reciprocal communication between peripheral sensory neurons and mast cells, thereby sustaining chronic neuroimmune inflammation and persistent pruritus.

## 4. The Mast Cell–Substance P Neuroimmune Axis

The communication between sensory neurons and mast cells represents a key mechanism underlying cutaneous neuroimmune inflammation. Rather than functioning as independent cellular compartments, these two systems establish a dynamic bidirectional signaling network that integrates environmental stimuli and neuroimmune and vascular responses. In this network, SP acts as a central molecular mediator linking neuronal excitation to mast cell activation, while mast cell-derived mediators subsequently reinforce neuronal sensitization and self-perpetuating inflammatory circuits [8,51].

Peripheral sensory neurons are activated by a broad spectrum of endogenous and exogenous stimuli, including mechanical injury, microbial products, allergens, inflammatory cytokines, thermal stress, and psychological stress. Activation of transient receptor potential (TRP) channels, particularly TRPV1 and TRPA1, promotes membrane depolarization and calcium influx, ultimately triggering the release of neuropeptides from peripheral nerve terminals [52]. SP represents one of the earliest and most potent initiators of cutaneous neuroimmune interactions because of its ability to rapidly influence epithelial, vascular, and immune compartments simultaneously [53].

Once released into the cutaneous microenvironment, SP exerts pleiotropic biological effects through two complementary receptor systems. Activation of NK1R on endothelial cells, keratinocytes, fibroblasts, leukocytes, and additional resident skin cells promotes vasodilation, increased vascular permeability, leukocyte recruitment, and transcriptional activation of pro-inflammatory genes [6]. In experimental studies using connective tissue mast cells purified from human skin, SP can activate MRGPRX2 and induce rapid degranulation independently of FcεRI-mediated signaling [23].

MRGPRX2- and FcεRI-mediated mast-cell activation also display distinct activation kinetics. In human skin mast cells, MRGPRX2-triggered degranulation develops very rapidly, with maximal responses observed within approximately 1 min, whereas FcεRI-mediated activation follows a slower and more sustained time course [23]. This temporal difference suggests that SP–MRGPRX2 signaling may contribute particularly to rapid neuroimmune responses, whereas FcεRI-mediated activation may be more relevant to sustained allergic inflammation, with cytokine production appearing to differ more quantitatively than qualitatively between the two pathways [54]. The coexistence of these parallel pathways enables SP to coordinate immediate responses while simultaneously amplifying mast cell effector functions, thereby linking neuronal activation with both innate and adaptive immune responses.

Unlike classical IgE-mediated hypersensitivity, this neuroimmune pathway does not require prior allergen sensitization. In murine models, neuronal stimulation can induce mast-cell activation through MrgprB2-dependent mechanisms [55], supporting a role for IgE-independent mast-cell activation in neurogenic inflammatory responses [56]. This concept has substantially expanded the traditional view of mast cell biology and supports the potential contribution of IgE-independent pathways to chronic neuroimmune inflammation [57].

Activated mast cells release an extensive repertoire of preformed and newly synthesized mediators that profoundly influence both immune and neuronal function [58]. Histamine, tryptase, chymase, prostaglandins, leukotrienes, TNF-α, IL-4, IL-6, IL-13, IL-31, nerve growth factor, and vascular endothelial growth factor collectively promote vascular leakage, inflammatory cell recruitment, tissue remodeling, and peripheral nerve sensitization [59]. Importantly, several of these mediators directly increase neuronal excitability, lowering the activation threshold of peripheral nociceptors and facilitating additional neuropeptide release [60,61].

One of the most distinctive characteristics of the mast cell–SP axis is its ability to generate self-sustaining feed-forward neuroimmune circuits. Rather than constituting a linear signaling cascade, neuroimmune communication within the skin is increasingly recognized as a dynamic amplification system in which neuronal and immune components continuously reinforce one another. The process is initiated by activation of peripheral sensory neurons, leading to local SP release within the dermis. Experimental and mechanistic evidence from human mast-cell systems and murine models indicates that SP can activate mast cells through NK1R-dependent pathways and, respectively, through human MRGPRX2 or its murine functional ortholog MrgprB2, promoting degranulation and the release of multiple inflammatory mediators [62,63]. These mediators, in turn, further sensitize adjacent sensory nerve endings through activation of histamine receptors, protease-activated receptors, cytokine receptors, and transient receptor potential channels. As neuronal activation thresholds progressively decrease, additional SP is released, thereby completing a positive feedback loop that perpetuates inflammation independently of the initial triggering stimulus [7].

This amplification mechanism provides a plausible explanation for the persistence of chronic pruritus observed in several inflammatory skin diseases, even after the initiating stimulus has resolved. Mast cells and sensory neurons may allow neuroimmune activation to persist, contributing to recurrent disease flares, sustained vascular dysfunction, and progressive tissue remodeling. Such loops also help explain why symptom severity often exceeds the degree of visible inflammation and why psychological stress frequently precipitates disease exacerbations [64].

Moreover, additional neuropeptides, including calcitonin gene-related peptide (CGRP), vasoactive intestinal peptide (VIP), and neurokinin A, may further modulate mast cell activation and neuronal excitability together with SP, highlighting the complexity of cutaneous neuroimmune communication [63,65]. Even so, current experimental evidence supports the SP–MRGPRX2 pathway as an important molecular interface linking peripheral sensory neurons with mast-cell effector functions [14,66].

**Figure 1:**
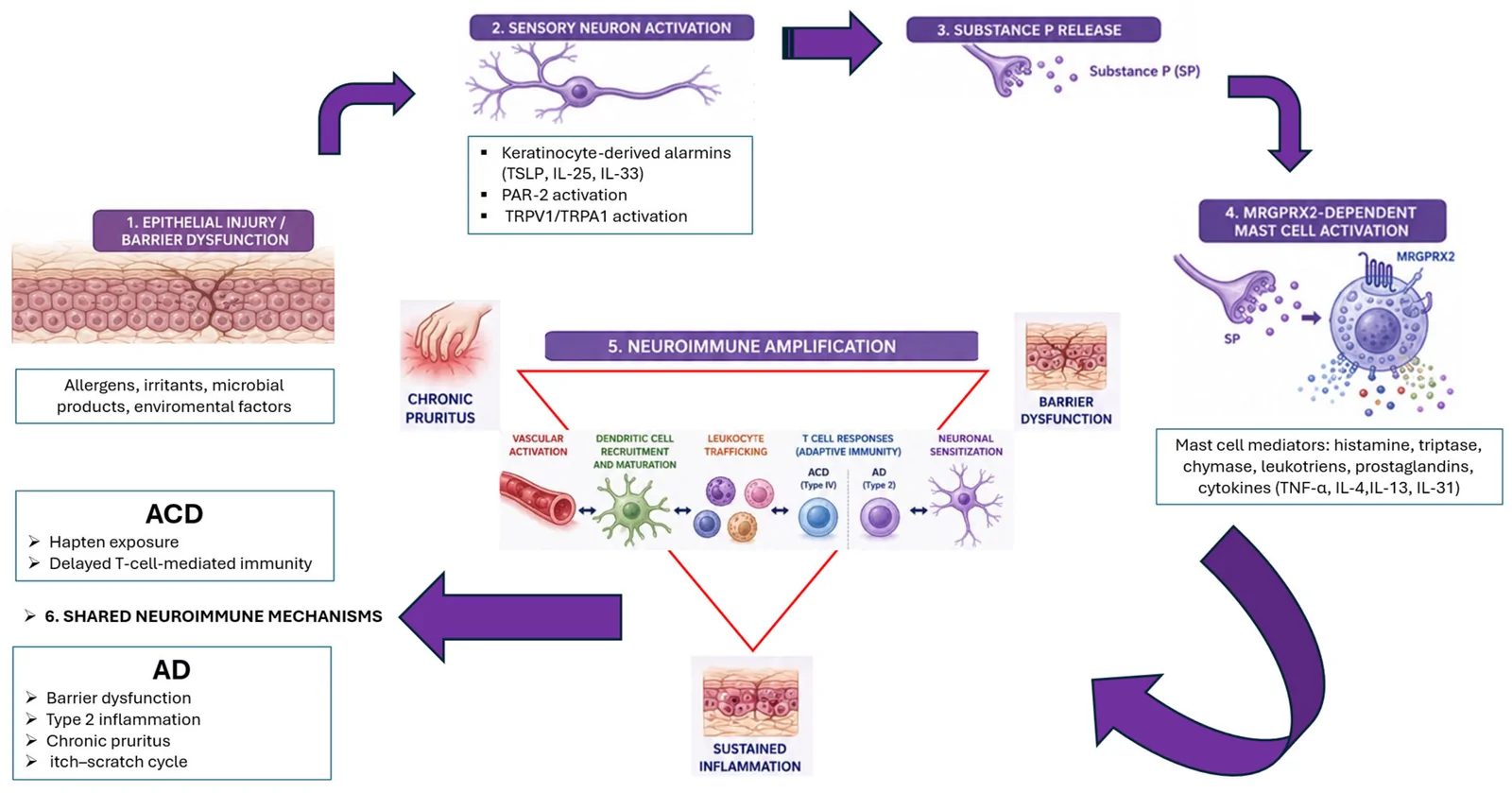
Neuroimmune amplification through the mast cell–substance P axis in allergic contact dermatitis and atopic dermatitis. Schematic representation of the shared neuroimmune mechanisms linking epithelial injury or barrier dysfunction to sensory neuron activation, substance P release, mast-cell activation, and downstream inflammatory amplification. Keratinocyte-derived mediators, PAR-2 activation, and TRPV1/TRPA1 signaling contribute to sensory neuron activation and SP release. SP may subsequently activate mast cells through MRGPRX2-dependent signaling, promoting rapid degranulation and mediator release. Mast-cell-derived mediators further enhance vascular activation, dendritic-cell recruitment and maturation, leukocyte trafficking, adaptive immune responses, neuronal sensitization, chronic pruritus, and barrier dysfunction, thereby contributing to sustained inflammation. Although these neuroimmune mechanisms are shared, their immunopathological context differs between the two diseases: in ACD, they operate alongside hapten-driven delayed T-cell-mediated immunity, whereas in AD they occur in the setting of barrier dysfunction, type 2 inflammation, chronic pruritus, and the itch–scratch cycle. Direct disease-specific evidence for the SP–MRGPRX2 pathway remains more limited in human ACD. TSLP, thymic stromal lymphopoietin; IL, interleukin; PAR-2, protease-activated receptor-2; TRPV1, transient receptor potential vanilloid 1; TRPA1, transient receptor potential ankyrin 1; SP, substance P; MRGPRX2, Mas-related G protein-coupled receptor X2; ACD, allergic contact dermatitis; AD, atopic dermatitis. Created in BioRender. Ginaldi, L. (2026) https://BioRender.com/sew1oth.

Mast cells and sensory neurons therefore constitute an integrated functional unit, rather than acting as isolated components, and jointly contribute to chronic pruritus, inflammation, and tissue remodeling. This concept substantially shifts the traditional interpretation of allergic skin diseases, suggesting that persistent inflammation is maintained not only by immune dysregulation but also by continuous reciprocal signaling between these two compartments [67]. Additionally, neuroinflammation may function as a self-amplifying neuroimmune network long after the initiating trigger has resolved, in chronic inflammation of both the skin [68] and the central nervous system, where analogous feed-forward mechanisms involving microglia have been described [69,70,71,72]. Elucidating the molecular basis of these circuits may improve our understanding of the heterogeneous clinical manifestations of allergic and inflammatory skin diseases and support the development of therapeutic strategies specifically targeting neuroimmune pathways rather than individual downstream inflammatory mediators (Figure 1).

## 5. The Mast Cell–Substance P Axis in Allergic Skin Diseases

The pathogenic relevance of the neuroimmune circuitry described above becomes particularly evident in allergic skin diseases, where persistent signaling between sensory neurons and mast cells contributes to the main cellular, molecular and clinical features. Allergic skin diseases encompass a heterogeneous group of chronic inflammatory disorders, including atopic dermatitis, chronic urticaria, allergic contact dermatitis, and other pruritic dermatoses, which collectively represent a major global health burden owing to their high prevalence, recurrent course, and substantial impact on patients’ quality of life [73,74,75]. Despite differing in their immunological endotypes, clinical presentation, and predominant inflammatory pathways, these disorders share common neuroimmune mechanisms that actively contribute to disease initiation, persistence, and exacerbation [76] through a complex pathogenetic interplay. In particular, SP acts as a key molecular mediator linking neuronal activation to mast cell degranulation and inflammatory responses, whereas mast cell-derived mediators further promote neuronal sensitization, establishing self-amplifying feedback loops and sustaining the main pathophysiological domains of this interaction previously introduced, namely chronic inflammation, pruritus, vascular alterations, and tissue remodeling. These features appear to constitute a common biological substrate underlying several allergic dermatoses, although the relative contribution of the mast cell–SP axis varies depending on the predominant immune endotype and the inflammatory microenvironment of each disease [77].

### 5.1. Allergic Contact Dermatitis: A Mechanistically Informative Model of Cutaneous Neuroimmune Activation

Among allergic skin disorders, ACD may represent a valuable model for investigating neuroimmune inflammation because its immunopathogenesis follows a well-defined pathogenic sequence linking tissue injury, innate immunity, adaptive immune activation, and neuronal signaling, with experimental murine studies also supporting a modulatory role of sensory neurons in contact hypersensitivity [78,79,80]. Unlike IgE-mediated allergic diseases, ACD is a type IV delayed hypersensitivity reaction initiated by repeated exposure to low-molecular-weight haptens that penetrate the epidermal barrier and bind endogenous proteins, generating neoantigens and inducing antigen-specific T-cell responses [81,82,83]. Importantly, neuronal activation and mast cell responses seem to occur during the earliest phases of cutaneous inflammation, preceding the full development of adaptive immune responses [84].

Following hapten penetration, keratinocytes constitute the first cellular sensors of tissue injury. Through the release of damage-associated molecular patterns (DAMPs), reactive oxygen species (ROS), alarmins, and pro-inflammatory cytokines, keratinocytes activate resident dendritic cells, Langerhans cells, endothelial cells, and adjacent sensory nerve endings [85,86]. Simultaneously, tissue injury and inflammatory mediators stimulate peptidergic C-fibers and thinly myelinated Aδ fibers to release neuropeptides, particularly SP and calcitonin gene-related peptide (CGRP), thereby initiating the neurogenic component of the inflammatory response [65]. This early neuronal response does not replace classical immune pathways but provides an immediate amplification system that bridges epithelial damage with innate immune activation.

SP released from activated sensory neurons may engage both NK1R expressed by structural cells and MRGPRX2 expressed by connective tissue mast cells. Activation of MRGPRX2 induces rapid, IgE-independent mast cell degranulation, leading to the release of tryptase, histamine, lipid mediators, cytokines, and chemokines that increase vascular permeability, facilitate plasma extravasation, promote leukocyte recruitment, and enhance dendritic cell migration and maturation. Consequently, mast cells may function not only as effector cells of inflammation but also as neuroimmune amplifiers linking early neuronal activation with the subsequent development of antigen-specific adaptive immune responses [22]. However, the direct contribution of the SP–MRGPRX2 pathway to human ACD remains incompletely established and is currently supported mainly by experimental evidence.

SP and CGRP further amplify inflammation by promoting endothelial activation and immune cell trafficking. Conversely, mast cell-derived mediators actively sensitize peripheral sensory neurons. Interestingly, tryptase activates protease-activated receptor-2 (PAR-2) expressed on sensory nerve endings, triggering additional release of SP and CGRP and reinforcing neuronal excitability [87,88]. This loop establishes a positive feedback mechanism in which neuronal activation continuously promotes mast cell degranulation, while mast cell mediators progressively lower the activation threshold of sensory neurons, thereby sustaining neurogenic inflammation independently of the initiating allergen [7]. In contrast, signaling through the cannabinoid receptors CB1 and CB2 provides an endogenous counter-regulatory mechanism within this circuit, with CB1 primarily reducing sensory neuron excitability and CB2 limiting mast cell activation and cutaneous inflammation [89,90].

This bidirectional interaction also extends beyond the sensitization phase. In murine models, repeated allergen exposure has been shown to reinforce neuroimmune signaling, promoting sustained SP release, recurrent mast cell activation, persistent dendritic-cell stimulation, and continuous recruitment of inflammatory cells [91,92]. Accordingly, neuronal and immune activation establish reciprocal circuits that amplify local inflammatory responses and are thought to contribute to the persistence and chronic evolution of ACD [61,67].

In addition to amplifying inflammation, the mast cell–SP axis plays a central role in chronic pruritus, one of the most disabling clinical manifestations of ACD. Persistent activation of sensory nerve fibers together with sustained release of mast cell-derived mediators, including tryptase, cytokines, leukotrienes, and additional neuropeptides, sensitizes peripheral nociceptors through PAR-2, TRPV1, TRPA1, and related signaling pathways. Therefore, itch perception becomes increasingly dissociated from the extent of visible skin inflammation, providing a mechanistic explanation for the persistence of severe pruritus despite apparent clinical improvement of cutaneous lesions. These histamine-independent neuroimmune pathways may also explain the limited efficacy of conventional antihistamines in a subset of patients with chronic allergic skin diseases [93].

Thus, ACD may represent an informative model for understanding how the mast cell–SP axis orchestrates cutaneous neuroimmune inflammation. Its sequential immunopathogenesis, from epithelial injury and innate immune activation to sensory neuron stimulation, mast cell degranulation, dendritic cell activation, antigen-specific T-cell responses, and chronic neuroimmune amplification, provides a useful perspective for interpreting the shared pathogenic mechanisms operating across allergic skin diseases.

Even though the relative contribution of this pathway varies according to the predominant immunological endotype of each disorder, ACD illustrates particularly well how reciprocal communication between sensory neurons and mast cells may contribute to the transition from an initially protective inflammatory response to persistent inflammation.

### 5.2. Atopic Dermatitis: Neuroimmune Amplification in Chronic Type 2 Inflammation

ACD may provide a useful model for understanding the establishment of the mast cell–SP neuroimmune axis, but AD represents the condition in which these pathways have been most extensively investigated [94,95]. Indeed, much of our current understanding of neuroimmune crosstalk derives from studies performed in AD, where chronic pruritus, persistent inflammation, and barrier dysfunction have revealed the central contribution of sensory neuron–immune cell interactions [96]. Building upon the pathogenic background described for ACD, many of the same neuroimmune mechanisms can be recognized in AD, although they operate within a profoundly different immunological context. While in ACD delayed type IV hypersensitivity and hapten-specific T-cell responses initiate neuroimmune activation, AD is primarily driven by chronic type 2 inflammation, epidermal barrier dysfunction, and genetic susceptibility, together with environmental factors and microbial dysbiosis [97,98,99,100]. Consequently, the mast cell–SP axis should not be regarded as an alternative pathogenic pathway but rather as an amplifying neuroimmune network that integrates with Th2 immunity, perpetuates chronic inflammation, and contributes to the remarkable clinical heterogeneity of AD [101].

In the pathogenesis of AD, barrier disruption facilitates the penetration of allergens, irritants, and microbial products, leading to keratinocyte activation and the release of epithelial-derived alarmins, including thymic stromal lymphopoietin (TSLP), IL-25, and IL-33 [102,103,104]. These mediators initiate and amplify type 2 immune responses while simultaneously promoting the activation of cutaneous sensory neurons and resident mast cells, thereby establishing an early neuroimmune dialogue within the skin [105,106].

The evidence for an association between SP and disease severity is inconsistent, but across the available clinical studies, this neuropeptide shows a clearer relationship with pruritus. Ewa Teresiak-Mikołajczak et al. found that plasma SP concentration correlated with pruritus intensity in 75 AD patients but reported no correlation between SP levels and the Scoring Atopic Dermatitis (SCORAD) severity index [107]. In contrast, Toyoda et al. reported significant correlations between plasma SP and disease activity using different severity scoring systems in 52 AD patients [108]. These discrepancies regarding severity may reflect differences in measurement approaches, as well as differences between plasma and skin expression and in the scoring systems used.

Overall, the pruritus association appears more consistently supported, with multiple studies demonstrating this relationship. Experimental evidence from NC/Nga mice, a murine model of AD, has also demonstrated alterations in cutaneous neuropeptide concentrations [109]. Increased SP expression has been shown in both lesional skin and peripheral nerve fibers of patients with AD, suggesting that persistent neuronal activation may represent a hallmark of chronic disease [110]. Although evidence supports increased SP expression in lesional AD skin and nerve fibers, the available data do not definitively establish whether neuronal activation is primary or secondary to inflammation [65,109]. Following its release, SP activates NK1R-expressing structural and immune cells while directly stimulating connective tissue mast cells through MRGPRX2, thereby initiating rapid degranulation and the release of multiple pro-inflammatory mediators [47,63]. Activated mast cells subsequently release pruritogenic mediators and cytokines such as IL-31, all of which contribute to neuronal sensitization and chronic itch. In an IL-13-induced murine model of chronic AD, TRPA1 was shown to contribute to pruritic responses, supporting the involvement of sensory ion channels in neuroimmune itch amplification [93]. In addition, mast cell-derived proteases and inflammatory mediators further impair epidermal barrier integrity, enhancing transepidermal water loss and facilitating additional allergen penetration. Together, barrier dysfunction, neuronal activation, and mast cell responses become tightly interconnected, generating a self-perpetuating inflammatory network that persists independently of the initial triggering event [66].

A particularly relevant feature of AD is the establishment of the itch–scratch cycle, one of the clearest examples of feed-forward neuroimmune amplification in clinical dermatology. Persistent itch induces repetitive scratching, resulting in mechanical disruption of the epidermis, increased release of epithelial alarmins, and further activation of sensory neurons and mast cells. Far from representing a simple behavioral consequence of itching, scratching actively contributes to disease progression by reinforcing the interplay between nerves and immune cells. This positive feedback explains why pruritus often persists despite partial control of visible skin inflammation and why disease flares frequently occur in the absence of new allergen exposure [111,112], despite the well-established association of AD with sensitization to common allergens [113].

Recent evidence also suggests that chronic type 2 inflammation further enhances neuroimmune communication. Cytokines such as IL-4 and IL-13 not only regulate adaptive immune responses but also directly increase neuronal responsiveness by sensitizing peripheral sensory neurons to pruritogenic stimuli [67]. Several studies support these mechanisms. Mithilesh Kumar Jha et al. demonstrated that IL-4 and IL-13 promote dorsal root ganglion sensory neuron growth in both mouse and human cultures, with effects similar to or greater than IL-31, and that IL-13 and IL-31 elicit acute scratching behavior in mice [114]. L. Oetjen et al. established that type 2 cytokines directly activate sensory neurons through neuronal IL-4Rα and JAK1 signaling, with chronic itch dependent on these pathways [115]. M. Furue et al. identified IL-31 as a “cardinal pruritogenic cytokine” produced by type 2 T cells, noting that IL-4 and IL-13 amplify IL-31-mediated sensory nerve signaling [116]. Simultaneously, IL-31, produced by activated T helper 2 cells and other immune populations, acts directly on cutaneous sensory nerves, further amplifying itch perception and SP release [93]. These observations indicate that adaptive immunity and neuroimmune signaling should not be considered independent pathogenic pathways but rather complementary components of a highly integrated inflammatory network.

Within this integrated neuroimmune network, mast cells emerge as dynamic amplifiers of chronic inflammation rather than passive effector cells. Their strategic localization adjacent to sensory nerve endings allows continuous bidirectional communication with the peripheral nervous system, while their broad secretory repertoire sustains vascular dysfunction, immune cell recruitment, tissue remodeling, and neuronal sensitization. Persistent activation of the mast cell–SP axis therefore contributes not only to inflammatory maintenance but also to symptom chronicity and disease heterogeneity.

From a translational perspective, recognition of these mechanisms provides a biological rationale for therapeutic approaches targeting neuroimmune communication in addition to conventional immunomodulation. While biologic agents directed against IL-4, IL-13, and IL-31 have significantly improved disease control [117,118], increasing understanding of SP signaling, MRGPRX2 activation, and mast cell–neuron interactions suggests that combined modulation of immune and neuroimmune pathways may represent an important strategy for patients with persistent pruritus or incomplete clinical responses [46,94,110]. Future therapeutic approaches may benefit from integrating immunological and neurobiological mechanisms to achieve more comprehensive and durable disease control.

Despite these shared neuroimmune mechanisms, their pathogenic context differs substantially between ACD and AD. In ACD, neuroimmune amplification operates alongside a predominantly delayed, hapten-specific T-cell response, whereas in AD it operates in the setting of chronic type 2 inflammation, epidermal barrier dysfunction, and persistent pruritus. Moreover, direct disease-specific evidence for the SP–MRGPRX2 axis is currently more limited in human ACD, while neuroimmune alterations have been more extensively investigated in AD. Thus, the two diseases provide complementary settings in which similar mast cell–neuronal interactions are integrated into distinct immunological backgrounds, as summarized in Table 1.

## 6. Discussion

Inflammatory dermatitis has traditionally been interpreted according to the adaptive immune mechanisms that define each disease. ACD is classically regarded as the prototype of delayed type IV hypersensitivity, whereas AD represents the paradigm of chronic type 2 inflammation associated with epidermal barrier dysfunction. Although these models remain fundamental for understanding disease initiation, they do not entirely explain several clinical features shared by both disorders, including persistent pruritus, stress-induced exacerbations, chronic disease progression, and the frequently poor correlation between the intensity of inflammation and symptom severity [119,120,121].

The evidence reviewed here supports the concept that these classical immunological paradigms may be complemented by an integrated neuroimmune perspective. The functional interplay between peripheral sensory neurons and mast cells appears to act as a dynamic amplification system sustaining immune activation once inflammatory responses have been initiated, more than a simple consequence of inflammation [122].

### 6.1. Beyond Immediate Hypersensitivity: Neuroimmune Amplification of Delayed T-Cell Responses

One of the most intriguing aspects emerging from the available evidence concerns the biological role of MRGPRX2. Traditionally, this receptor has been investigated almost exclusively in the context of immediate IgE-independent hypersensitivity reactions, including pseudoallergic drug reactions, chronic spontaneous urticaria, angioedema, and mast-cell activation disorders. Hence, MRGPRX2 has generally been viewed as a receptor primarily associated with rapid mast-cell degranulation [24,123].

The present review proposes a broader interpretation. Unlike previous neuroimmune models, which have largely considered atopic dermatitis as the reference disease for studying cutaneous neuroimmune interactions [124,125], we considered ACD as a useful mechanistic starting point. This choice reflects the observation that ACD offers a relatively well-defined temporal sequence linking epithelial injury, innate immune activation, sensory neuron stimulation, SP release, mast-cell activation, dendritic-cell maturation, and ultimately antigen-specific T-cell responses.

Within this background, activation of the SP–MRGPRX2 axis may act as a neuroimmune amplifier alongside classical delayed hypersensitivity. Experimental studies have shown that SP can activate human mast cells through MRGPRX2, supporting a potential contribution of this pathway to ACD. SP released by activated sensory neurons may activate mast cells through MRGPRX2, promoting degranulation and the release of mediators involved in vascular activation, cytokine production, dendritic-cell recruitment, leukocyte trafficking, and sustained neuronal sensitization. These events may reinforce and sustain T-cell-mediated inflammation without replacing its fundamental immunological basis. However, direct disease-specific evidence establishing the SP–MRGPRX2 pathway in human ACD remains limited, and its proposed contribution is currently supported largely by mechanistic studies of human mast cells and by experimental models of cutaneous neuroinflammation.

Several related processes can also be recognized in AD. In contrast to ACD, neuroimmune amplification develops within a considerably more complex biological environment characterized by type 2 inflammation, epithelial barrier dysfunction, microbial dysbiosis, and chronic itch. From this perspective, ACD may provide a useful setting for examining the contribution of the mast cell–SP axis to chronic inflammatory dermatitis.

This interpretation also reshapes the biological role traditionally attributed to mast cells. These cells are increasingly recognized as dynamic regulators of neuronal and immune signaling, with functions extending well beyond their role as immediate effector cells, amplifying adaptive immune responses through continuous bidirectional communication with peripheral sensory neurons.

Pruritus provides a clinically relevant example of neuroimmune amplification. Following mast-cell activation, released tryptase can activate protease-activated receptor-2 (PAR-2) on cutaneous sensory nerve endings, increasing neuronal excitability and facilitating the release of neuropeptides such as SP and CGRP. These mediators may in turn reinforce mast-cell activation and local inflammatory signaling, progressively lowering the threshold for further neuronal activation and contributing to the persistence of itch during chronic inflammation. This reciprocal mast cell–nerve interaction may therefore contribute to persistent and clinically relevant pruritus in chronic inflammatory dermatitis [6,7,64].

### 6.2. Shared Neuroimmune Mechanisms Across Inflammatory Skin Diseases

The evidence reviewed here highlights the neuroimmune mechanisms underlying allergic contact dermatitis and atopic dermatitis. However, the coordinated interplay among SP signaling, MRGPRX2 activation, mast-cell degranulation, PAR-2 activation, and peripheral sensory neurons suggests that related neuroimmune amplification mechanisms may operate across inflammatory skin diseases despite their remarkably diverse adaptive immune profiles [14,126].

This concept may help explain why related mechanisms extend beyond these two disorders and why diseases driven by distinct immunological pathways frequently share common clinical manifestations, including persistent pruritus, neurogenic inflammation, stress-related disease exacerbations, and incomplete responses to conventional anti-inflammatory or anti-histaminic therapies [4,127].

Thus, neuroimmune circuits may represent shared biological amplifiers superimposed on disease-specific immune pathways, although their relevance outside ACD and AD requires further investigation.

Growing evidence supports the involvement of neuroimmune signaling in chronic spontaneous urticaria, angioedema, and non-IgE-mediated hypersensitivity reactions, particularly through MRGPRX2-dependent mast-cell activation [128,129,130]. Neurogenic inflammation has also been proposed as a potential component of brain–gut–skin communication [131,132]. In this context, shared pathways involving neural signaling, SP, and mast cells have been hypothesized to contribute to the reported association between chronic spontaneous urticaria and gastroesophageal reflux disease (GERD) [133,134]. However, direct evidence for a causal neuroimmune link between these conditions remains limited, and this possibility should currently be regarded as hypothesis-generating.

Severe T-cell-mediated cutaneous adverse drug reactions, including DRESS syndrome and Stevens–Johnson syndrome, represent another area in which the potential contribution of neuroimmune mechanisms remains largely unexplored [135]. Their pathogenesis is primarily driven by complex immune and cytotoxic pathways, and direct evidence supporting a role for the mast cell–SP axis is currently lacking [136,137,138]. Nevertheless, the occurrence of prominent cutaneous symptoms, including pruritus in some patients, suggests that neuroimmune signaling may warrant investigation as a possible modifier of symptom expression rather than as a primary pathogenic mechanism.

From a translational perspective, recognizing the existence of a neuroimmune amplification system naturally generates new therapeutic hypotheses. Beyond conventional immune-directed therapies, several emerging approaches may be of interest, including modulation of the SP/NK1R pathway [139], selective inhibition of MRGPRX2-dependent mast-cell activation [46,57,140], and pharmacological targeting of the endocannabinoid system, including topical non-THC cannabinoids such as palmitoylethanolamine (PEA) and cannabidiol [141,142]. These strategies may simultaneously influence neuronal activation, mast-cell responsiveness, neurogenic inflammation, and chronic pruritus. Likewise, modulation of transient receptor potential (TRP) channels may further contribute to interrupting the reciprocal communication between sensory neurons and mast cells [143,144,145]. These interventions should be regarded as complementary to established immunomodulatory therapies, targeting the neuroimmune amplification mechanisms that contribute to disease persistence.

### 6.3. Limitations and Future Perspectives

Growing evidence supports the role of neuroimmune interactions in allergic and inflammatory diseases. Nevertheless, several important limitations should be acknowledged.

A major translational limitation concerns the substantial reliance of mechanistic studies on animal models. In particular, murine mast cells express MrgprB2, the functional ortholog of human MRGPRX2, but the two receptors differ in ligand recognition and pharmacological responsiveness. Accordingly, findings obtained in MrgprB2-based models cannot be directly extrapolated to human MRGPRX2 biology, and validation in human mast cells, skin tissue, and disease-specific clinical settings remains essential before these pathways can be considered reliable therapeutic targets.

This review focused specifically on allergic contact dermatitis and atopic dermatitis because these conditions represent complementary models of adaptive immune activation, providing the opportunity to examine neuroimmune mechanisms in both delayed T-cell-mediated and type 2 inflammatory settings. As a result, several disorders in which neuroimmune signaling is increasingly recognized, including chronic spontaneous urticaria, angioedema, psoriasis, rosacea, and severe cutaneous adverse drug reactions, were beyond the scope of this review. Nevertheless, this focused perspective offers a conceptual basis that may be evaluated across a broader spectrum of inflammatory skin diseases.

Similarly, this review was not intended to provide a comprehensive overview of therapeutic interventions. Instead, the therapeutic concepts discussed here are presented as a natural extension of the mechanistic and pharmacodynamic insights developed throughout the review.

Finally, current knowledge also remains constrained by the available evidence. Much of the current evidence derives from experimental models or small observational studies, while robust longitudinal and interventional clinical data remain limited. In addition, considerable methodological heterogeneity exists across studies, particularly regarding the assessment of SP, whose short biological half-life, rapid enzymatic degradation, differences in sample processing, and variability in analytical techniques make the comparison of circulating and tissue levels challenging. Consequently, the absence of standardized neuroimmune biomarkers limits the ability to establish consistent correlations between mediator expression, disease activity, and therapeutic response. Future studies integrating tissue-based analyses, longitudinal sampling, and standardized biomarker assessment will be important for better defining the clinical relevance of the mast cell–SP axis and identifying reproducible neuroimmune endotypes that may guide precision medicine approaches.

## 7. Conclusions

Recent advances in cutaneous neuroimmunology have fundamentally reshaped our understanding of inflammatory skin diseases, demonstrating that the nervous and immune systems function as a highly integrated network within the skin. Reciprocal interactions among sensory neurons, mast cells, structural skin cells, and adaptive immune cells drive the initiation, amplification, and persistence of cutaneous inflammation. Neuroimmune signaling therefore extends the classical immunological paradigm by providing an additional regulatory dimension that helps explain disease heterogeneity, chronicity, and variability in therapeutic responses.

Using allergic contact dermatitis as a useful model for examining neuroimmune amplification, this review illustrates how neuroimmune pathways amplify T cell-mediated inflammation, while atopic dermatitis demonstrates how the same mechanisms operate within a complex type 2 inflammatory environment. Across these distinct immunological settings, the mast cell–SP axis, together with MRGPRX2-dependent signaling, emerges as an important neuroimmune interface linking neuronal activation to sustained immune dysregulation, chronic inflammation, and persistent pruritus.

Current evidence supports an important role for neuroimmune signaling in chronic inflammatory skin diseases, although its relative contribution and disease-specific relevance remain to be fully defined. Future studies integrating mechanistic investigations with tissue-based analyses, longitudinal sampling, and standardized neuroimmune biomarker assessment will be essential for validating disease-specific neuroimmune endotypes and translating these advances into precision medicine.

Collectively, these findings support neuroimmune communication as an important contributor to disease persistence and symptom expression, while its impact on therapeutic responsiveness requires further clinical validation. A deeper integration of neurobiology with immunology may therefore refine current concepts of allergic skin diseases and support the development of more individualized therapeutic strategies. In clinical practice, a better characterization of neuroimmune mechanisms may ultimately support more precise patient stratification, particularly in relation to persistent pruritus and symptom burden, and contribute to more individualized management of pruritic inflammatory dermatoses.

## Figures and Tables

**Table 1:** Key comparative neuroimmune mechanisms of the mast cell–substance P axis in allergic contact dermatitis and atopic dermatitis.

Neuroimmune Mechanism	Biological Mechanism	Pathophysiological Implication	ACD	AD	Key References
Substance P signaling	Activates NK1R- and MRGPRX2-dependent neuroimmune pathways	Links sensory neuron activation to mast cell-driven inflammation and chronic pruritus	Proposed amplifier of hapten-driven, T-cell-mediated inflammation; direct human disease-specific evidence remains limited	Associated with chronic pruritus and neuroimmune signaling in a type 2 inflammatory setting	[37,40,46,65]
MRGPRX2 activation	Triggers IgE-independent mast cell degranulation in response to neuropeptides	Provides an alternative pathway for neurogenic inflammation in allergic skin diseases	Potential IgE-independent mast-cell amplification pathway; mainly supported by mechanistic and experimental evidence	Potential contributor to mast-cell–neuronal signaling within chronic type 2 inflammation	[9,13,14,46]
Neuron–mast cell crosstalk	Establishes bidirectional communication between sensory neurons and mast cells	Sustains inflammatory and pruritogenic signaling through reciprocal activation	May amplify delayed hypersensitivity responses	Contributes to chronic itch and persistent neuroimmune activation	[7,8,34,64]
Histamine-independent Itch	Activates non-histaminergic neuronal pathways involving PAR-2, TRPV1, and TRPA1	Contributes to persistent pruritus and variable response to antihistamines	May contribute to persistent pruritus through PAR-2/TRP signaling	Prominent component of chronic pruritus and the itch–scratch cycle	[7,57,63,67]
Neuroimmune amplification	Feed-forward interactions perpetuate neuron–mast cell signaling	Favors disease persistence, chronic inflammation, and recurrent flares	Amplifies hapten-specific delayed T-cell-mediated inflammation	Develops within barrier dysfunction and chronic type 2 inflammation	[7,8,61,64]

Shared and disease-specific features are highlighted. Key molecular mediators in allergic contact dermatitis (ACD) and atopic dermatitis (AD) include neurokinin-1 receptor (NK1R), Mas-related G protein-coupled receptor X2 (MRGPRX2), protease-activated receptor-2 (PAR-2), transient receptor potential vanilloid 1 (TRPV1), and transient receptor potential ankyrin 1 (TRPA1).

## Data Availability

No new data were created or analyzed in this study. Data sharing is not applicable to this article.
